# Simulated Microgravity Causes Delayed Platelet Activation and Downregulates Acid-Sensing Ion Channel 1/2 Protein Expression

**DOI:** 10.3390/biomedicines13122860

**Published:** 2025-11-24

**Authors:** Niharika Bala, Ling Yu, Neil S. Harris, Faisal Mukhtar, Abdel A. Alli

**Affiliations:** 1 Division of Nephrology, Hypertension, and Renal Transplantation, Department of Medicine, University of Florida, Gainesville, FL 32610, USA; 2 Department of Physiology and Aging, University of Florida, Gainesville, FL 32610, USA; 3 Department of Pathology, Immunology, and Laboratory Medicine, University of Florida Health, Gainesville, FL 32610, USA; harris@pathology.ufl.edu (N.S.H.);; 4 Department of Pediatrics, College of Medicine, University of Florida, Gainesville, FL 32610, USA

**Keywords:** simulated microgravity, ASIC, platelets, membrane fluidity, sphingomyelin

## Abstract

**Background:** Microgravity is a physical force that affects cellular functions, including gene expression, cellular differentiation, proliferation, and signal transduction. Ion channels play an important role in ionic permeability and cell physiology. In addition, ion channels have been shown to contribute to volume regulation, fluid homeostasis, blood pressure regulation, mechanosensation, and cell migration. The lipid composition and fluidity of the plasma membrane of various cell types contribute to the regulation of ion channels. We hypothesized that protein expression of acid-sensing ion channels (ASICs) is decreased while membrane fluidity is increased, leading to delayed activation of human platelets subject to microgravity conditions. **Methods and Results:** Platelets were maintained in simulated microgravity conditions using the rotating wall vessel method. Thromboelastography analysis showed there is a delay in platelet activation in human platelet samples subject to simulated microgravity conditions compared to normal gravity for 5 days at 37 °C. Western blotting and immunofluorescence microscopy studies showed that ASIC1/2 proteins are significantly downregulated in human platelets subject to the same simulated microgravity conditions. In addition, membrane fluidity was increased while sphingomyelin concentration was decreased in human platelets subject to simulated microgravity compared to normal gravity conditions. **Conclusions:** Taken together, the data from this study suggest that simulated microgravity delays platelet activation in human platelets in a mechanism presumably involving a decrease in ASIC1/2 protein expression and sphingomyelin plasma membrane concentration.

## 1. Introduction

Microgravity is a physical force that affects several cellular functions, including cellular differentiation, proliferation, migration, signaling, and protein expression. As space technology advances, a growing number of studies have investigated the effects of microgravity on human health on Earth. The effects of microgravity on various types of specialized cells, including platelets, have been previously investigated [1].

Platelets play important roles in wound healing [2], angiogenesis [3,4], inflammatory processes [5,6], and cancer progression [7,8]. The expression of various ion channels is thought to mediate the function of platelets. Transient receptor potential (TRP) proteins are ion channels classified into subfamilies, including the canonical TRPC channels, polycystin TRPP channels, melastatin TRPM channels, mucolipin TRPML channels, and vanilloid TRPV channels. Human platelets have been reported to express TRPC1, 3, 4, 5, and 6 [9]. Liu et al. reported that TRPC6 expression is increased in platelets from patients with type 2 diabetes mellitus compared to nondiabetic individuals [10].

Several calcium-sensitive ion channels have been reported to be expressed in human platelets. Human platelets have been reported to express the calcium release-activated calcium channel Orai1 and the calcium-sensitive intermediate conductance potassium channel KCa3.1 [11]. Studies using pharmacological inhibitors suggested these channels contribute to platelet migration [11]. Another study reported pannexin-1 channels are expressed at the surface of human platelets and pharmacological inhibition of these channels reduced calcium influx, ATP release, thrombus formation, and platelet aggregation [12].

Multiple studies have reported that the highly selective cation channel epithelial sodium channel (ENaC) is expressed in platelets. Studies involving the pharmacological inhibition of ENaC using amiloride suggested this channel is involved in granule secretion, platelet migration, and platelet collagen activation [13]. Other studies have reported that ENaC is overexpressed in platelets from hypertensive patients [14] and ENaC expression in platelets is a biomarker for arterial hypertension [13]. Acid-sensing ion channels (ASICs) are known to associate with ENaC subunits, but the expression of ASIC in platelets has not been investigated.

Stimulated microgravity has been shown to attenuate the activity of the L-type voltage-sensitive calcium channel [15]. Simulated microgravity was previously shown to activate the T-type Ca_V_3.1 channel in rat cerebral vascular smooth muscle cells [16]. Another study reported that the activity of large-conductance Ca^2+^-activated K^+^ (BK_Ca_) and K_V_ channel activity in vascular smooth muscle cells is decreased in cerebral arteries after 1 week of simulated microgravity in cerebral arteries and increased after 1 week and 4 weeks of simulated microgravity in small mesenteric arteries. For volt-age-gated K^+^ (K_V_) channels, the activity was decreased after 4 weeks of simulated mi-crogravity for the cerebral arteries and increased after 1 week and 4 weeks of simulated microgravity in small mesenteric arteries. [17]. Another study reported downregulation of TRPC6 in response to simulated microgravity [18]. Kamkin et al. showed simulated microgravity altered the gene transcript levels of several mechanically gated channels including TRPV2, TRPM7, TRPP1/2, TMEM63A, TMEM36B and Piezo1 as well as several mechanosensitive channels including Ca_V_1.2, K2P2.1, Na_V_1.5, K2P3.1, Kir6.1, Kir6.2, and K_V_7.1 in isolated rat ventricular cardiomyocytes [19].

Although specific ion channels have been shown to play various roles in human platelets, and simulated microgravity has been shown to suppress specific ion channels, the regulation of specific ion channels including ASIC/ENaC in human platelets under microgravity conditions has not been studied. Here, we utilized a rotary bioreactor to simulate microgravity conditions on earth and investigated the effect of clotting parameters in human platelets and the regulation of ASIC 1/2 and ENaC alpha protein expression. In addition, we measured changes in sphingomyelin concentration and membrane fluidity as a potential mechanism for the regulation of ASIC1/2 protein expression and platelet activation.

## 2. Materials and Methods

### 2.1. Platelets from Human Volunteers

Human platelets were obtained from the Blood Bank at the University of Florida. Platelets were prepared by the platelet-rich plasma (PRP) method through the centrifugation of whole blood, followed by a further concentration step after the red cells were separated. The citrated anticoagulant (acid citrate dextrose) was added for platelet recovery, function, and survival. Platelets were suspended in 45–65 mL of plasma to maintain the pH at 6.2 or higher for 5 days. There is approximately 1 to 1.5 billion total platelets in 1 mL of PRP prepared using this method. Platelet products were stored at room temperature (20–24 °C) with continuous gentle agitation.

### 2.2. Simulated Microgravity

For each human donor sample, 2–3 mL platelets were placed in a vessel of a rotating wall vessel bioreactor (Synthecon, Inc.; Houston, TX, USA), while the same volume of a second batch of the sample platelets was placed only in the vessel (normal gravity). Rotation was carried out at 17 rpm. Both batches of platelets were incubated at 37 °C for 5 days. The rotating wall vessel is a cylindrical culture vessel that rotates horizontally to provide an optimized environment for suspension cell culture. Its solid body rotation around a horizontal axis significantly reduces shear forces and turbulence compared to traditional stirred bioreactors, thereby lowering mechanical stress on cells. This rotation also simulates microgravity conditions, closely mimicking the natural low-shear environment cells experience during normal development in living organisms. A Rotating Wall Vessel (RWV) bioreactor is widely reported to generate a residual gravity environment of approximately 10^−3^ *g* through a balance of gravitational, centrifugal, and fluid dynamic forces acting on the cells. Prior studies have quantitatively characterized and validated this microgravity level, confirming the RWV setup as an established ground-based analog for biological microgravity research [20,21].

### 2.3. BCA Protein Assay

A bicinchoninic acid (BCA) assay was performed to determine total protein concentrations from lysed human platelets. Briefly, 9 standards were prepared from serial dilutions of a stock solution of 2 mg/mL of bovine serum albumin (Millipore Sigma, St. Louis, MO, USA). A solution of BCA reagent A and B (ThermoFisher, Waltham, MA, USA) was added to each standard and sample in a 96-well plate. A linear regression line was used to calculate the protein concentration of each sample after reading the plate at 570 nm.

### 2.4. Membrane Fluidity Assay

Membrane fluidity in human platelets cultured under normal gravity or simulated microgravity was measured using a commercial assay (Abcam, Waltham, MA, USA; ab189819) while following the manufacturer’s instructions. Briefly, platelets from both groups were first plated in a 96-well plate. A labeling solution was prepared by diluting 5 µM pyrenedecanoic acid (PDA) fluorescent lipid reagent and supplementing with Pluronic F-127 at 0.08% final concentration. Platelets were incubated with this labeling solution for 1 h at room temperature in the dark with gentle agitation to ensure uniform labeling. After incubation, unincorporated PDA was removed by washing cells twice. The labeled platelets were then resuspended or maintained in fresh buffer. Fluorescence readings were taken at the monomer emission (~400 nm) and excimer emission (~470 nm) after excitation at 350 nm to calculate the ratio of excimer to monomer fluorescence, which quantitatively reflects membrane fluidity.

### 2.5. SDS-PAGE, Western Blotting, and Densitometric Analysis

Platelet samples harvested from microgravity and control (normal gravity) conditions were centrifuged at 6000 rpm for 10 min. The resulting pellets were resuspended in EasyPrep buffer (ThermoFisher Scientific, Waltham, MA, USA) and sonicated for 5 s. A BCA protein assay was performed to determine the total protein concentration for each lysate. Fifty micrograms of each lysate were loaded into separate wells of 12-well 4–20% Tris-HCl polyacrylamide gels (Thermo Fisher, USA) and resolved in SDS buffer (Bio-Rad, Hercules, CA, USA) at 200 volts for 1 h. Following electrophoresis, proteins were transferred onto nitrocellulose membranes (ThermoFisher Scientific) using Towbin buffer at 100 volts for 45 min. The membranes were stained with 1X Ponceau S for 5 min and subsequently washed with type 1 water to visualize the bands. After washing the membranes three times with 1X TBS for 3 min each, they were blocked in 5% nonfat dry milk for 1 h at room temperature. The membranes were then incubated overnight at 4 °C with the ASIC1 primary antibody (S271-44; Stress Marq, Victoria, BC, Canada) or ASIC2 primary antibody (PA5-87945; Invitrogen, Waltham, MA, USA) (Table 1), each at a 1:1000 dilution in 5% BSA 1X TBS solution. The following day, the membranes were washed again three times with 1X TBS for 3 min each and then incubated with the secondary antibody (1:3000 dilution in blocking solution) for 1 h. Finally, the membranes were incubated with ECL solution (BioRad) for 7 min and imaged using an iBright imager (ThermoFisher Scientific). The intensity of the protein bands of interest was normalized to Ponceau S staining. Band intensities for both Ponceau S and the proteins of interest were quantified using ImageJ 1.54g (NIH) [22]. Data plotting and statistical analysis were performed using Sigma Plot 15.0 software, with a *p*-value of less than 0.05 considered statistically significant.

### 2.6. Immunofluorescence Microscopy

Platelet samples harvested from simulated microgravity and normal gravity control conditions were centrifuged at 6000 rpm for 10 min. The pellets were suspended in Hank’s Balanced Salt Solution (HBSS) (Gibco, Grand Island, NY, USA). Next, 35 mm glass-bottom dishes (Matek; Ashland, MA, USA) were coated with a cell attachment factor and incubated at room temperature for 10 min. A 200 µL drop of the suspended platelets was added to the center of the dish and allowed to air dry for 30 min at room temperature. Afterwards, the cells were fixed using a 1:1 solution of methanol and acetone for 10 min at −20 °C. After two washes with 1X PBS, 2.5% horse serum was added as a blocking solution and incubated at room temperature for 20 min. The blocking solution was then aspirated, and a 1:200 dilution of the primary antibody was added and incubated for 45 min at room temperature. After two washes with 1X PBS, a secondary antibody was added to each dish and incubated for an additional 45 min at room temperature. Finally, the dishes were washed with 1X PBS, and antifade mounting media containing DAPI (Vector, Newark, CA, USA) was added, and the dishes were cover-slipped and imaged using an Olympus BX41 (Center Valley, PA, USA) fluorescence microscope.

### 2.7. Thromboelastography

Thromboelastograph or TEG analysis was performed as a measure of whole blood coagulation that considered both the coagulation factor activities (including that of fibrinogen) as well as the contribution of platelets to this process. To perform the test, a fresh citrated whole blood specimen was mixed with kaolin, an activator of the intrinsic pathway of coagulation. Next, 340 µL of this mixture was added to a disposable sample cup containing 20 µL of CaCl_2_ to reverse the effects of the citrate, and the cup was inserted into a reaction chamber and held at 37 °C. This step placed a detection pin in the liquid specimen. The cup was then rotated slowly clockwise and then anticlockwise by the analyzer. The cup oscillated 4°45′ in either direction every 4.5 s. As coagulation proceeded, the specimen became more viscous, which produced torsion on the pin that generated a signal via a torsion wire connected to a detector, thereby indicating the increased viscosity. The TEG tracings initially show a straight line that then splits apart as the specimen clots (becomes more viscous). These split lines diverge until the distance between the lines reaches a stable maximum amplitude. Table 2 shows the list of parameters acquired on these platelets from the TEG analysis.

### 2.8. Statistical Analysis

A one-way ANOVA was used to test significance between multiple groups, and Student’s *t*-test was used to test significance between 2 groups using SigmaPlot 15. All data are presented as the mean ± SEM.

## 3. Results

### 3.1. Simulated Microgravity Causes a Delay in the Activation of Platelets from Human Donors

**Figure 1:**
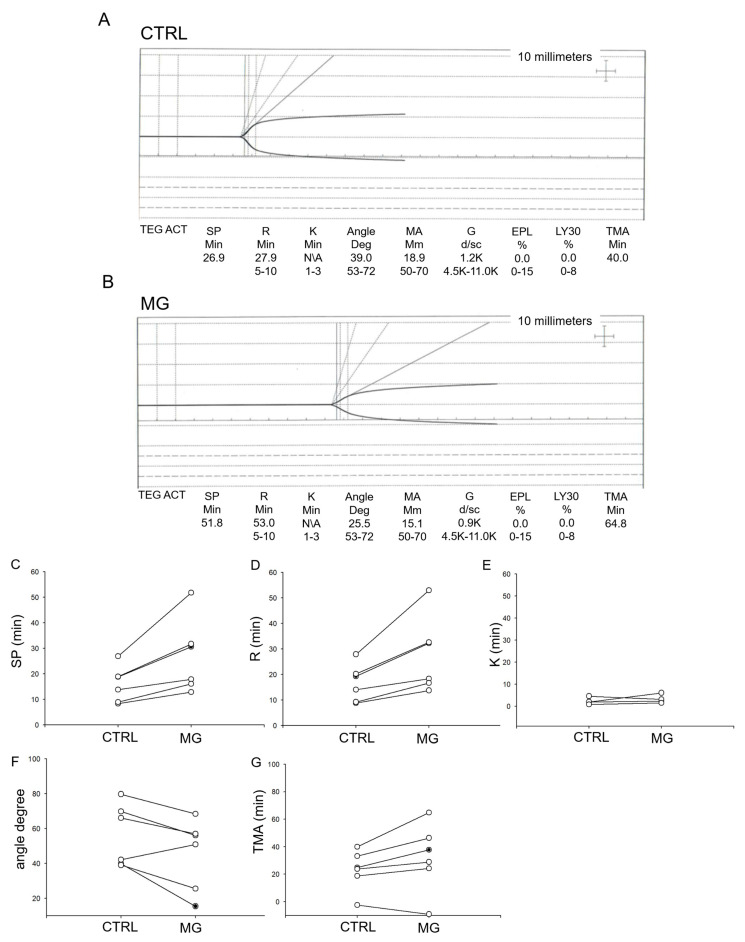
Thromboelastograph (TEG) analysis of human platelets subject to normal gravity control (CTRL) or simulated microgravity (MG) conditions. n = 6 donors. (**A**) Representative thromboelastograph from human platelets subject to normal gravity conditions, (**B**) Representative thromboelastograph from human platelets subject to microgravity conditions, (**C**) split time, (**D**) the latency time (R time), (**E**) time between the trace reaching 2 mm and going up to 20 mm (K time), (**F**) Plot upslope angle (**G**) time needed to reach maximum MA (TMA).

Human platelets from normal healthy donors (n = 5) were subject to normal gravity or simulated microgravity (via a rotating bioreactor) conditions for 5 days. The platelets were collected and then used for TEG analysis to measure various parameters. As shown in TEG tracings in human platelets subject to simulated microgravity conditions showed delayed platelet activation (Figure 1).

### 3.2. ASIC1 Protein Expression Is Decreased in Human Platelets Subject to Simulated Microgravity Conditions

**Figure 2:**
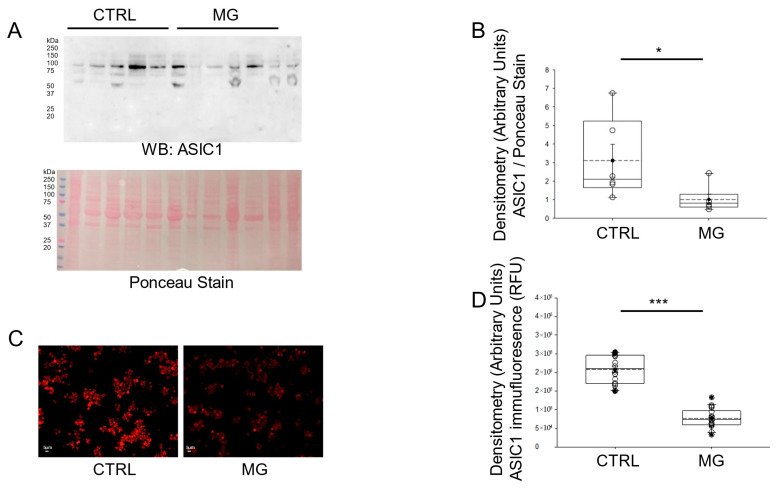
Western blot and densitometric analysis of ASIC1 protein expression in human platelets subject to normal gravity control (CTRL) or simulated microgravity (MG) conditions. (**A**) Western blot of ASIC1 protein expression in lysates from platelets of healthy donors subject to normal gravity control (CTRL) or simulated microgravity (MG) conditions for 5 days. Platelets from n = 6 donors. (**B**) Densitometric Analysis of the immunoreactive bands for ASIC1 in panel (**A**). (**C**) Representative Immunofluorescence microscopy images of ASIC1 protein expression in fixed platelets from healthy donors subject to normal gravity control (CTRL) or simulated microgravity (MG) conditions for 5 days. (**D**) Densitometric analysis of 18 platelets from each human donor subject to normal gravity control (CTRL) or simulated microgravity (MG) conditions for 5 days. Platelets from n = 3 donors. * represents a *p*-value < 0.05, *** represents a *p*-value < 0.001. The dashed line within the box plots represents the median.

Ion-channel-dependent physiological functions at the cellular level are affected by gravity [23]. Both Western blotting and immunofluorescence microscopy analysis showed a significant decrease in ASIC1 protein expression in human platelets maintained under simulated microgravity conditions compared to normal gravity conditions (Figure 2).

### 3.3. ASIC2 Protein Expression Is Decreased in Human Platelets Subject to Simulated Microgravity Conditions

**Figure 3:**
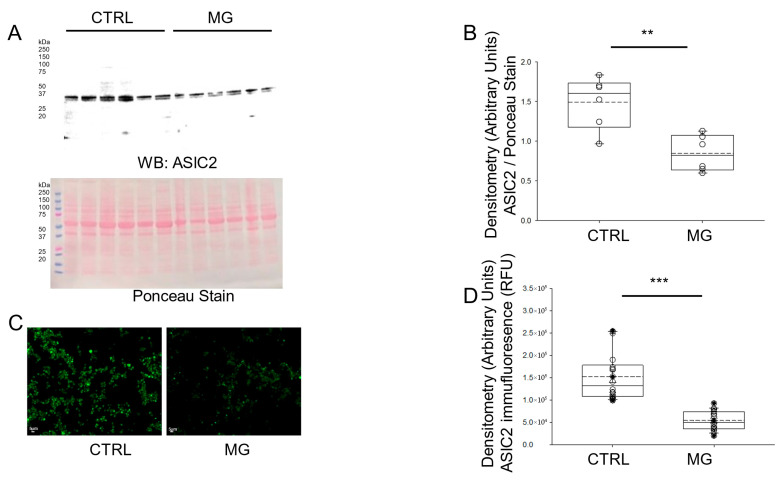
Western blot and densitometric analysis of ASIC2 protein expression in human platelets subject to normal gravity control (CTRL) or simulated microgravity (MG) conditions. (**A**) Western blot of ASIC2 protein expression in lysates from platelets of healthy donors subject to normal gravity control (CTRL) or microgravity (MG) conditions for 5 days. Platelets from n = 6 donors. (**B**) Densitometric Analysis of the immunoreactive bands for ASIC2 in panel (**A**). (**C**) Representative Immunofluorescence microscopy of ASIC2 protein expression in fixed platelets from healthy donors subject to normal gravity control (CTRL) or simulated microgravity (MG) conditions for 5 days. (**D**) Densitometric analysis of 18 platelets from each human donor subject to normal gravity control (CTRL) or simulated microgravity (MG) conditions for 5 days. Platelets from n = 3 donors. ** represents a *p*-value < 0.01, *** represents a *p*-value < 0.001. The dashed line within the box plots represents the median.

Since the protein expression of ASIC1 was found to be decreased in platelets subject to simulated microgravity, we next investigated whether ASIC2 protein expression was also affected. Like ASIC1, Western blotting and immunofluorescence microscopy analysis showed that the protein expression for ASIC2 was significantly less in human platelets maintained under simulated microgravity conditions compared to normal gravity conditions (Figure 3).

### 3.4. ENaC Alpha Subunit Protein Expression Is Comparable in Human Platelets Subject to Simulated Microgravity and Normal Gravity Conditions

**Figure 4:**
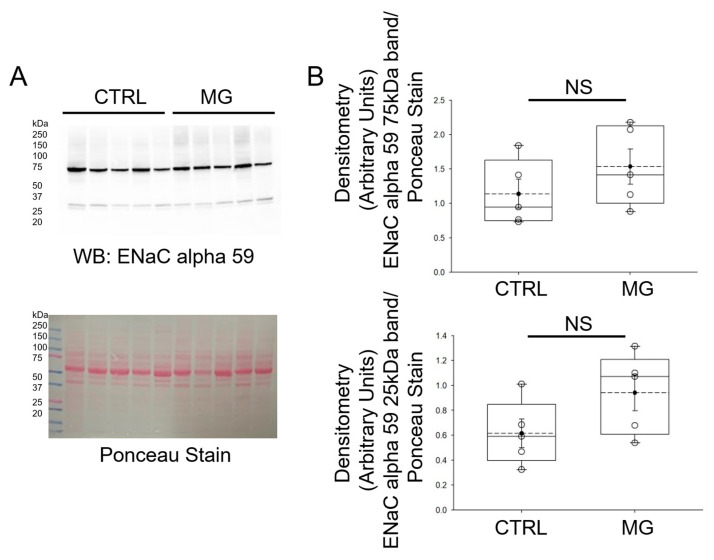
Western blot and densitometric analysis of ENaC alpha protein expression in human platelets subject to normal gravity control (CTRL) or simulated microgravity (MG) conditions. (**A**) Western blot of ENaC protein expression in lysates from platelets of healthy donors subject to normal gravity control (CTRL) or simulated microgravity (MG) conditions for 5 days. Platelets from n = 5 donors. (**B**) Densitometric Analysis of the immunoreactive bands for ENaC alpha in panel A. NS represents no significance between the groups. The dashed line within the box plots represents the median.

Like ASICs, ENaC is also a member of the superfamily of ion channels that play a key role in chemosensation, mechanosensation, and the regulation of blood volume and blood pressure. Also, ENaC and ASICs have been shown to form hybrid channels [24,25]. Therefore, we measured whether protein expression of the alpha subunit of ENaC is affected by simulated microgravity conditions. As shown in Figure 4, ENaC alpha protein expression was comparable between the two groups of human platelets maintained under normal gravity and simulated microgravity conditions.

### 3.5. Membrane Fluidity Is Increased in Human Platelets Subject to Simulated Microgravity Conditions

**Figure 5:**
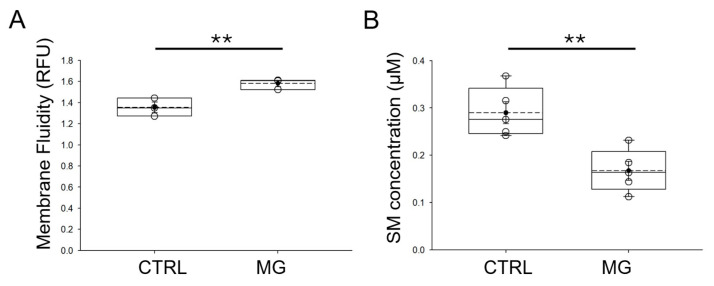
Relative membrane fluidity and sphingomyelin concentrations in human platelets subjected to microgravity and normal gravity conditions. (**A**) Summary plot of relative membrane fluidity in human platelets subject to simulated microgravity (MG) and normal gravity (CTRL) conditions. (**B**) Summary plot of relative sphingomyelin concentration in human platelets subject to simulated microgravity and normal gravity conditions. CTRL refers to control normal gravity conditions. n = 5 samples per group. ** represents a *p*-value < 0.01. The dashed line within the box plots represents the median.

The properties of lipid membranes are thought to directly affect the expression and activity of ion channels. Since both ASIC [26] and ENaC are regulated by lipids [27,28], we next investigated whether simulated microgravity conditions affect the membrane fluidity of human platelets. Human platelets from normal healthy donors were subject to normal gravity or microgravity conditions for 5 days before membrane fluidity was assessed. As shown in Figure 5A, human platelets maintained under simulated microgravity conditions had an increase in membrane fluidity when compared to human platelets maintained under normal gravity conditions. We also measured sphingomyelin concentrations in human platelets from each group. As shown in Figure 5B, sphingomyelin concentration was lower in human platelets subject to simulated microgravity conditions compared to normal gravity control conditions.

## 4. Discussion

The goal of this study was to investigate how microgravity affects protein expression of specific ion channels and platelet activation. This study investigates whether simulated microgravity causes a delay in platelet activation and investigates differences in ASIC1/2 and ENaC alpha protein expression in human platelets subject to simulated microgravity compared to normal gravity conditions. Finally, this study investigates whether simulated microgravity affects sphingomyelin enrichment and membrane fluidity in human platelets.

Platelets have been shown to express several different types of ion channels, and these channels are thought to regulate specific functions. The inwardly rectifying potassium GIRK channels and the large conductance Ca^2+^-activated K^+^ channel KCa1.1 have been reported to contribute to collagen-induced adhesion and pro-coagulation activity [29]. Human platelets have been shown to express chloride channels that contribute to membrane depolarization and platelet activation [30]. The adenosine triphosphate-gated cation channel, P2X1 receptor, was found to regulate platelet thrombosis [31].

Microgravity has been proposed to have beneficial and detrimental effects. The ability of microgravity to induce apoptosis in cancer cells has been recently reviewed [32]. Studies have shown microgravity regulates the expression of proteins involved in tumor growth and invasion. One study reported microgravity increases the expression of TIMP-1, TIMP-2, MMP-2, MMP-9 in H1703 and A549 cells [33].

To maintain rigor and reproducibility, we used platelets from each donor that were still viable after being commissioned for research use. Second, we took precautions not to introduce air bubbles in the vessel while culturing each batch of platelets to avoid creating a volatile environment. Third, we subjected each batch of platelets from each human donor to the same amount of time in the bioreactor. Finally, we used validated antibodies in our protein biochemistry experiments and confirmed the changes in ASIC1/2 protein expression by both Western blotting and immunofluorescence microscopy (Table 2).

Microgravity exposure during spaceflight may impact human physiology in many ways. It leads to adaptive cardiac atrophy, reduction in left ventricular mass and cardiac contractility, reduced circulating blood volume, and reduced diastolic blood pressure. All of these changes lead to a reduced peak exercise performance both in-flight and in the immediate period after return. Plasma and red blood cell volume also decrease during spaceflight, with an increased risk of degradation of hemoglobin and increased incidence of hemolytic anemia. Impairments in the immune system are also common, with differential production of inflammatory cytokines and increased risk of activation of viruses [34]. At a cellular level, spaceflight induces apoptosis in lymphocytes through pathways such as the FAS/APO-1 (sFas) [35], calcium-dependent 5-LOX activation [36,37], damaged mitochondrial membranes and cytochrome c release, and caspase activation. Some studies have shown that cells tend to downregulate genes associated with cell death and inhibition of cell cycling in an effort to adapt to or survive the microgravity environment [38].

Microgravity induces significant changes at cellular and molecular levels, affecting cell structure and function across various biological systems. Key mechanisms involve alterations in cytoskeletal organization and signaling pathways such as PI3K and Wnt/β-catenin, which are crucial for cell growth and differentiation [39]. Electrophysiological experiments in microgravity have demonstrated that ion-channel-dependent physiological processes are altered under mechanical unloading, and membrane properties can directly affect ion channel function [23]. Studies show that microgravity increases membrane fluidity, which decreases the open-state probability of ion channels; membrane viscosity and lateral pressure are gravity-dependent parameters directly influencing ion channel activity [40].

We hypothesized that the effects of weightlessness would change the properties of the platelet cell membrane, including changes in membrane fluidity and ASIC1 and ASIC2 protein expression. Data from this study show an increase in membrane fluidity in human platelets subject to microgravity conditions compared to normal gravity conditions. Consistent with these results, sphingomyelin concentration was found to be decreased in human platelets subject to microgravity compared to normal gravity. These results are consistent with results from a study by Pedrera et al. that show a high content of sphingomyelin induces lower lateral diffusion and/or liquid-condensed phases [41]. Published studies have reported that altered membrane fluidity is associated with changes in signal transduction of human platelets [42] and megakaryoblasts [43] during pathophysiology. Multiple studies have demonstrated that membrane fluidity is directly affected by gravity conditions, with microgravity generally increasing membrane fluidity compared to normal gravity. One group showed that membrane fluidity changes are a fundamental mechanism through which gravity interacts with cellular function and ion channel properties [44]. Furthermore, Kohn et al. found that the integration of lidocaine into cellular membranes and the gravity-dependence of this process further highlight the extent to which membrane fluidity is altered in microgravity environments [45]. These findings indicate that pharmacological responses and cellular signaling pathways may be profoundly influenced during spaceflight and other microgravity exposures.

There are some limitations of the current study. First, simulated microgravity from rotating bioreactors such as rotating wall vessels, 2D clinostats, or the random positioning machine that are commonly used to create a simulated microgravity environment, does not exactly recapitulate the zero-gravity found in space [46]. Instead, it allows us to model similar conditions and investigate various aspects of human platelet function. Recent studies have shown that simulated microgravity platforms, such as fast-rotating clinostats or random positioning machines, can introduce significant fluid motion and shear stress within the cell culture environment, causing biological responses that are not directly attributable to microgravity itself. For example, Mansour and Berwanger et al. demonstrated that cellular effects observed in clinostat experiments may often result from rotation-induced fluid shear rather than the intended reduction in gravitational vector, and are therefore artifacts rather than true microgravity effects [47]. Simulated microgravity using ground-based devices such as the rotating wall vessel provides valuable insights, but it has inherent limitations that must be considered when interpreting results. The mechanical forces generated by rotation, including residual shear stress and centrifugal forces, can introduce artifacts that differ from true microgravity experienced in spaceflight [48]. Additionally, simulation models may not fully replicate the complex multi-factorial environment of real microgravity. This necessitates careful interpretation of results to distinguish microgravity-specific effects from those caused by the culture system itself [49]. These limitations highlight the need for caution when extrapolating findings from simulated microgravity to actual spaceflight conditions.

The rotational motion of the fluid allowed the human platelets in suspension to be in a simulated free-fall over time. Second, our study was limited to measuring changes in the protein expression of ASIC1/2 in human platelets exposed to simulated microgravity or normal conditions. We did not measure the activity of these ASIC proteins or the mechanisms that regulate their function.

ASIC1/2 and ENaC protein expressions have been investigated by our group and others in the context of serving as biomarkers for disease pathophysiology. For example, ENaC alpha and ASIC1/2 were both found to be expressed in human pheochromocytoma wildtype cells and mutant cells with a knockdown of succinate dehydrogenase subunit B (SDHB), but there were significantly lower levels of the cleaved 60 kDa form of ENaC in SDHB KD cells [50]. Another study showed that high ENaC alpha subunit mRNA expression correlates with less proliferative and less aggressive breast cancer phenotypes, while decreased ENaC alpha subunit expression increases breast cancer cell proliferation [51]. Although data from this current study shows a significant decrease in ASIC1/2 protein expression, but not ENaC alpha protein expression in human platelets subject to microgravity conditions for 5 days, further studies are needed to determine whether the decrease in ASIC1/2 protein expression is directly associated with a delay in platelet activation or other aspects of platelet dysfunction. Other future directions include investigating the effects of microgravity on platelets from young and old human donors, since it has been shown that microgravity induces cellular senescence [52,53] and endothelial dysfunction [54]. These studies will investigate whether microgravity augments oxidative stress in platelets from older compared to younger donors. In addition, we plan to explore the potential for pharmacological modulation of ASICs to counteract the effects of microgravity on platelet function. This could lead to therapeutic strategies for maintaining platelet function during space missions. Also, the functional consequences of ASIC1/2 down-regulation on platelet activity, including aggregation, secretion, and interaction with other blood components, may be investigated in future studies. This could help elucidate the physiological impact of microgravity on hemostasis.

## 5. Conclusions

Simulated microgravity resulted in a significant delay in human platelet activation, accompanied by downregulation of ASIC1 and ASIC2 protein expression. Additionally, platelets had increased membrane fluidity due to reduced sphingomyelin levels. Notably, ENaC alpha subunit protein expression remained unchanged under these conditions, suggesting a specific regulatory impact of microgravity on ASICs in human platelets. These findings highlight a novel mechanism by which microgravity may impair platelet function, with implications for hemostatic balance during spaceflight and related applications.

## Figures and Tables

**Table 1:** List of antibodies used in this study.

Primary Antibody	Company	Catalog Number
ASIC 1	Invitrogen, Waltham, MA, USA	pa587945
ASIC 2	Stress Marq, Victoria, BC, Canada	s271-44
ENaC alpha 59	James A. Haley Veterans’ Hospital, Tampa, FL, USA	PMID: 22791334

**Table 2:** Definition of TEG parameters.

Parameter	Definition of Parameter
Clotting index (CI)	coagulation index for the specimen and is a function of the R time, K time and the MA. A hypercoagulable state is defined as CI greater than +3.0 and coagulopathy as CI less than −3.0.
Plot upslope angle	Overall speed of the clot formation
Maximum amplitude (MA)	measured in millimeters and is the greatest vertical amplitude of the split lines of the trace. It reflects clot strength and is a function both of platelet activity (about 80–85% of the MA) and fibrinogen activity (about 10–20% of the MA).
tMA	Time needed to reach maximum MA after start of the clot formation
G	Overall total clot strength or shear elastic modulus strength resulting from all coagulation interactions and is calculated from maximum amplitude (MA). Measured in dynes/cm^2^
K time	time between the trace reaching 2 mm and going up to 20 mm. The “alpha angle” is a slope drawn from the slope of the tracing from the R line to the K value. Both “K” and “alpha” are measurements of the kinetics of clot formation and are dependent on the fibrinogen activity of the specimen and to a certain degree, platelet function.
R time	the latency time from placing blood in the cup until the clot starts to form (taken as reaching a tracing amplitude of 2 mm). It is determined by the factors of the intrinsic and common coagulation pathways.

## Data Availability

Relevant datasets will be made available upon reasonable request after contacting the corresponding author.

## Abbreviations

The following abbreviations are used in this manuscript:

ASIC	Acid-sensing ion channels
BCA	bicinchoninic acid
CI	Clotting index
CTRL	control
ENaC	Epithelial sodium channel
MG	microgravity
PRP	Platelet-rich plasma
SDHB	succinate dehydrogenase subunit B
SM	sphingomyelin
TEG	Thromboelastograph
TRP	Transient receptor potential
